# A Novel Micro-Linear Vector for *In Vitro* and *In Vivo* Gene Delivery and Its Application for EBV Positive Tumors

**DOI:** 10.1371/journal.pone.0047159

**Published:** 2012-10-15

**Authors:** Hong-Sheng Wang, Zhuo-Jia Chen, Ge Zhang, Xue-Ling Ou, Xiang-Ling Yang, Chris K. C. Wong, John P. Giesy, Jun Du, Shou-Yi Chen

**Affiliations:** 1 Department of Microbial and Biochemical Pharmacy, School of Pharmaceutical Sciences, Sun Yat-sen University, Guangzhou, People’s Republic of China; 2 Institute of Clinical Pharmacology, School of Pharmaceutical Sciences, Sun Yat-sen University, Guangzhou, People’s Republic of China; 3 Department of Forensic Medicine, Zhongshan School of Medicine, Sun Yat-sen University, Guangzhou, Guangzhou, People’s Republic of China; 4 Department of Biology, Hong Kong Baptist University, Kowloon, Hong Kong SAR, People’s Republic of China; 5 Department of Veterinary Biomedical Sciences & Toxicological Center, University of Saskatchewan, Saskatchewan, Canada; 6 Guangzhou Center for Disease Control and Prevention, Guangzhou, China; Central China Normal University, China

## Abstract

**Background:**

The gene delivery vector for DNA-based therapy should ensure its transfection efficiency and safety for clinical application. The Micro-Linear vector (MiLV) was developed to improve the limitations of traditional vectors such as viral vectors and plasmids.

**Methods:**

The MiLV which contained only the gene expression cassette was amplified by polymerase chain reaction (PCR). Its cytotoxicity, transfection efficiency *in vitro* and *in vivo*, duration of expression, pro-inflammatory responses and potential application for Epstein-Barr virus (EBV) positive tumors were evaluated.

**Results:**

Transfection efficiency for exogenous genes transferred by MiLV was at least comparable with or even greater than their corresponding plasmids in eukaryotic cell lines. MiLV elevated the expression and prolonged the duration of genes *in vitro* and *in vivo* when compared with that of the plasmid. The *in vivo* pro-inflammatory response of MiLV group was lower than that of the plasmid group. The MEKK1 gene transferred by MiLV significantly elevated the sensitivity of B95-8 cells and transplanted tumor to the treatment of Ganciclovir (GCV) and sodium butyrate (NaB).

**Conclusions:**

The present study provides a safer, more efficient and stable MiLV gene delivery vector than plasmid. These advantages encourage further development and the preferential use of this novel vector type for clinical gene therapy studies.

## Introduction

An effective vector for gene delivery should afford satisfactory transfection efficiency while assuring safety clinical application [1]. Traditionally, plasmid and viral-based vectors are two commonly used vectors. However, limitations such as immunogenicity and cytotoxicity reduce the clinical viability of viral vectors [2]. While plasmid-based gene transfection is considered to be less toxic, the relatively small transfection efficiency and short duration of transgene expression of plasmids have limited the feasibility of this method in clinical applications [3]. Furthermore, the numerous CpG sequences contained in the plasmid backbone can cause immunotoxic effects, including the elimination of transfected cells by the host immune responses [4]. The immune responses caused by unmethylated CpG dinucleotide motifs can further decrease the efficiency of gene transfection [5]. Another significant disadvantage for propagation of plasmids in bacteria is that it contains bacterial remnants such as lipopolysaccharides (LPS) or endotoxins, which can cause adverse clinical effects [6]. Therefore, these traditional vectors should be improved before clinical translation.

Recently, several novel approaches have been used to improve the traditional vectors applied in gene therapy. For example, previous studies have revealed that minicircle DNA was less immunogenic, had greater *in vivo* diffusivity and more stability than conventional plasmids [7]–[9]. These characteristics are attributed to the smaller size of the molecules and little contamination with DNA sequences that originated in the bacteria. The minimalistic immunologically defined gene expression (MIDGE) vectors created by Witting and his colleagues [10] are linear molecules containing only a promoter, a target gene and an RNA stabilizing sequence, flanked by two short hairpin oligonucleotide sequences. Each MIDGE, particularly when it was conjugated with nuclear localization signal (NLS) peptides, has greater transfection efficiency as compared with its corresponding plasmid, both *in vitro* and *in vivo* [11]–[14]. However, production of MIDGEs are costly and time consuming, particularly for the conjugation of NLS peptides. PCR-amplified DNA fragments, used as a model for double-stranded synthetic genes in gene therapy, have been proven to be efficient for both *in vitro* and *in vivo* gene delivery [15], [16]. However, transfection efficiency of PCR-amplified DNA fragments is lower than that of plasmid, especially when the DNA fragment is delivered as cationic complexes [16]. This is likely due to the instability and poor rate of transcription of the DNA fragment when incorporated into cells. Therefore, all of these recently developed novel vectors require further modifications before they would be feasible for clinical application.

Here, we report the development of a novel linear DNA delivery vector. Briefly, the gene expression cassette was ligated with hairpin oligodeoxynucleotides (ODNs), amplified by PCR by use of a ligation mixture as the template, and purified by use of PCR cleanup kits. We have named the process the Micro-Linear Vector (MiLV). The capability and pro-inflammatory responses of the MiLV to deliver genes were investigated both *in vitro* and *in vivo*. As a proof of concept, the MiLV was evaluated as a vector in gene therapy for Epstein-Barr virus (EBV) positive cancer cells.

## Materials and Methods

### Cell Culture

Human embryo kidney cell line 293 (HEK 293), mouse embryonic fibroblast cell line NIH 3T3, human nasopharyngeal carcinoma line CNE2 and EBV-positive monkey (tamarin) lymphocyte cell line B95-8 were purchased from the American Type Culture Collection (ATCC) (Rockville, MD) and maintained in our laboratory. The HEK 293, NIH 3T3 and CNE2 cells were cultured in Dulbecco’s modified Eagle’s medium (DMEM) and B95-8 cell line was maintained in RPMI 1640 medium supplemented with 10% fetal bovine serum (FBS) and antibiotics at 37°C in a 5% CO_2_ incubator. The medium was replaced until cells became 80% confluent and then passaged using 0.25% trypsin/EDTA.

### Construction of *eGFP*-MiLV

**Figure 1:**
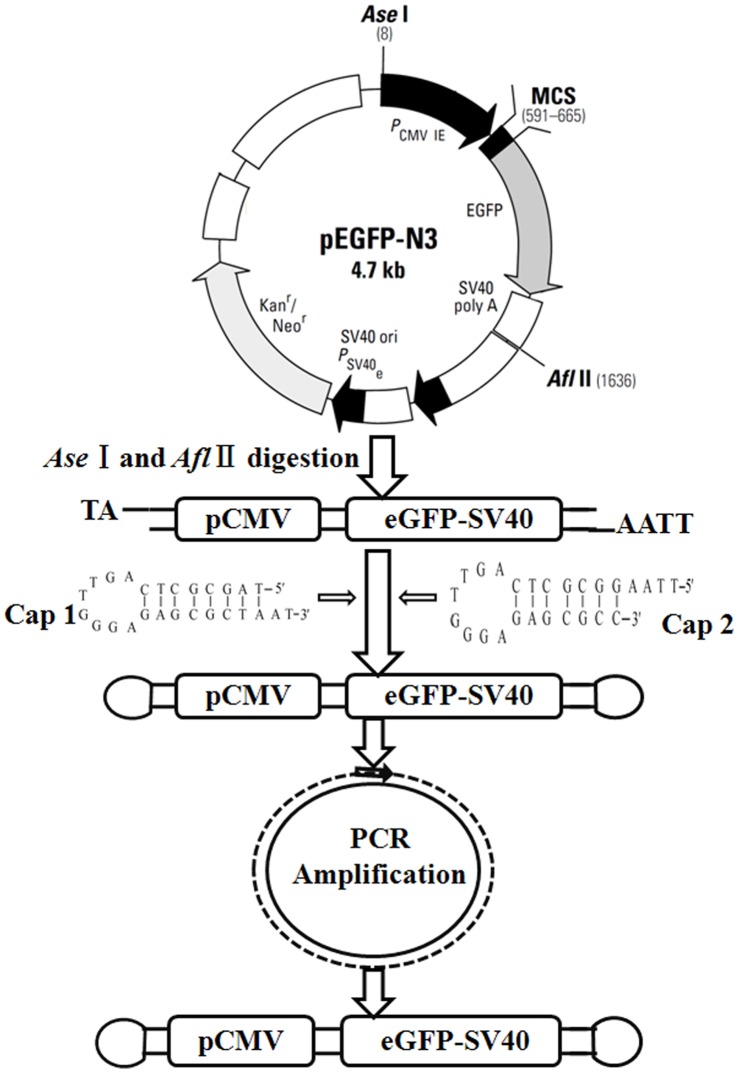
Construction of *eGFP*-MiLV. The eGFP expression cassette was digested using *AseI* and *Afl II* from *pEGFP-N3* plasmid. After the ligation of DNA fragment and ODN caps, the mixture was used as temple for PCR amplification. The *eGFP*-MiLV was purified by PCR cleanup kits and used for further *in vitro* and *in vivo* experiments.

Procedures for constructing the *eGFP*-MiLV are illustrated in Figure 1. Briefly, the *eGFP* expression cassette, including CMV promoter (pCMV), *eGFP* gene and RNA-stabilizing sequences (polyadenylic acid, SV40), was bi-digested by *AseI* and *Afl II* from *pEGFP-N3* plasmid (BD Biosciences Clontech, NJ) at 37°C for 4 h. Two ODN caps containing *AseI* and *Afl II* restriction enzyme site respectively were designed as structural analogues of tRNA’s D-loop in eukaryotic cells and synthesized by the Shanghai Sangon Company (Shanghai, China). The sequences of ODNs were: Cap 1∶5′-TAG CGC TCA GTT GGG AGA GCG CTA AT -3′; Cap 2∶5′-TTA AGG CGC TCA GTT GGG AGA GCG CC-3′. The *eGFP* expression cassette, Cap1, and Cap2 were mixed (molar ratio, 1∶10:10) and ligated overnight with T4 DNA ligase at 16°C. Then, 1 µl ligation mixture was used as the template to amplify *eGFP*- MiLV by PCR by use of a single primer (5′ ACA AGT TCA GCG TGT CCG 3′, annealing to 751 to 768 of the *eGFP* expression cassette) in a 50 µl reaction system. Amplification of *eGFP*-MiLV was performed by PCR under the following conditions: initial 94°C denaturation (2 min), followed by 35 cycles of three PCR steps (each cycle: 30 s at 94°C, 30 s at 54°C and 80 s at 72°C), and terminated with an extension prolongation for 5 min at 72°C. High success-rate DNA polymerase (Toyobo Co., Ltd., Osaka, Japan) was used to obtain sufficient DNA. After PCR, the products were purified by use of the E.Z.N.A.® Cycle-Pure Kit (Omega, USA). After the purified *eGFP*-MiLV was checked by DNA sequencing, it was used for cell transfection and intramuscular injection. To examine the *in vitro* resistance of MiLV to exonuclease, the *eGFP*-MiLV and *eGFP* fragment were incubated with Exonuclease III (Takara, Japan) at 37°C for 2 to 24 h and detected by 1% agarose gel electrophoresis.

### Construction of *pLMP1-MEKK1*-MiLV

The EBV genome was extracted from B95-8 cells using phenol/chloroform and purified by ethanol precipitation. The promoter of latent membrane protein 1 (*pLMP1*) was amplified from the EBV genome by PCR with the following primers: forward: 5′ GAC ATT AAT CTC AGG GCA GTG TGT CA G 3′; reverse: 5′ CCG CTC GAG TTG TGC AGA TTA CAC TGC 3′. The restriction enzyme sites of *AseI* and *XhoI* were contained at the 5′ and 3′ ends of *pLMP1*, respectively. The mitogen-activated protein kinase kinase (MEK) kinase 1 (*MEKK1*) gene with *XhoI* and *Afl II* restriction enzyme sites was amplified from the active form of pCMV/MEKK1Δ plasmid (Palo Alto, CA) with the following primers: forward: 5′ CCG CTC GAG CCA CCA TGG CGA TGT CAG CGT CTC 3′; reverse: 5′ CAG CTT AAG TTT ATT TGT GAA ATT TGT GAT GC 3′. Then, *pLMP1* and *MEKK1* were digested by *Xho* I at 37°C for 4 h and ligated by T4 DNA ligase at 16°C for 12 h. The *pLMP1-MEKK1* gene was amplified by PCR and ligated with two caps as mentioned above. The *MEKK1*-MiLV was amplified with the following primer: 5′ GAG TAA ATA CGG AGC TTT CAA GGA G 3′. The *MEKK1-*MiLV is approximately 1.5 kb, including the promoter (pLMP1) and MEKK1 gene.

### Cell Transfection

For transfection, cells were seeded into six-well plates at a density of 1×10^4^ cells/cm^2^, cultured for 24 h, and transfected with MiLV or plasmid DNA (0.1 µM DNA/cm^2^ per plasmid) with Lipofectamine reagent (Gibco BRL, Gaithersburg, MD) according to the manufacturer’s instructions. Briefly, cells were washed with phosphate-buffer saline (PBS) and before transfection cells resuspended in 800 µl culture medium without FBS or antibiotics. DNA and transfection reagent were diluted with 100 µl culture medium and incubated for 15 min at room temperature. Lipofectamine solution was added to the DNA solution and mixed, and then the mixture was incubated for a further 30 min (room temperature). The DNA/reagent mixture was added dropwise into the cell culture supernatant. Medium was replaced by 2 ml fresh medium supplemented with 10% FBS 2 h later. The transfection efficiency was defined as described previously [10] and evaluated 48 h after the transfection.

### Flow Cytometry

The green fluorescence in transfected cells was quantified by flow cytometry by use of an Epics XL (Coulter Immunotech, Hamburg, Germany). Cells were washed twice, resuspended with ice-cold PBS, and fixed with 70% ethanol. The fluorescence was measured by use of a 530-nm/30-nm band pass filter after illumination with an argon ion laser tuned at 488 nm. Cells transfected with transfection reagent served as the control. Magnitude of expression of GFP was reported as the percentage of GFP+ cells [10].

### Cell Proliferation and Cytotoxicity Assay

For cell proliferation assays, HEK 293 cells were inoculated into 6-well plates and incubated for 24 h before exposure to 1 µM plasmid or MiLV. Cells were harvested every 24 h, and then cell density was calculated by use of a hemacytometer. Cytotoxicity was determined by use of the MTT (3-(4, 5-dimethylthiazol-2-yl)-2, 5-diphenyltetrazolium bromide) (Sigma, St. Louis, MO) assay. Briefly, after being transfected by the *MEKK1-MiLV* or pCMV/MEKK1 plasmid for 24 h, B95-8 cells were treated with 1 mM NaB for 18 h followed by treatment with 100 µg/ml ganciclovir (GCV) for 3 or 6 days. MTT was then added to each well to make a final concentration of 0.5 g/L, and then cells were incubated for a further 4 h. Supernatant solutions were then aspirated, and the cells solubilized in 200 µL dimethyl sulfoxide (DMSO). Optical density was measured at 570 nm.

### *In vivo* Gene Transfer

BALB/c male mice (6–8 weeks, Experiment Animal Center of Sun Yat-sen University, Guangzhou, China) were reared and maintained under conventional breeding conditions with food and water *ad libitum*, on a 12∶12 h light: dark cycle. The experimental protocol was approved by the Ethics Committee for Animal Research at Sun Yat-sen University. Twenty micrograms *eGFP*-MiLV and 60 µg *pEGFP-N3* plasmid (equal molar) were packaged with Lipofectamine™ 2000 Reagent (1∶1.5, g/ml) in a total volume of 100 µl. The mixtures were injected intramuscularly within 5 s. The control group was injected with PBS. To investigate expression of GFP, in each group, at least one mouse was euthanized each week for a total of 8 weeks. Mice were euthanized by use of standard surgical procedures. Muscle was sectioned transversely (5 µm) with a Leica CM 1850 cryostat (Leica, Nussloch, Germany) maintained at −20°C. Sections were examined for expression of *GFP* by use of a laser scanning confocal microscopy. The calculation of fluorescence intensity were processed with previously published method [17] with slight modification. Briefly, both the normalized photon counts and the area of eGFP signals were quantified. Then we subtracted the photon counts/second/mm^2^ of region of interest (ROI) by the photon counts/second/mm^2^ of the eGFP− area and calculated the total photon counts of generated by eGFP+ cells by timing the normalized intensity with the area of eGFP+ region.

**Figure 2:**
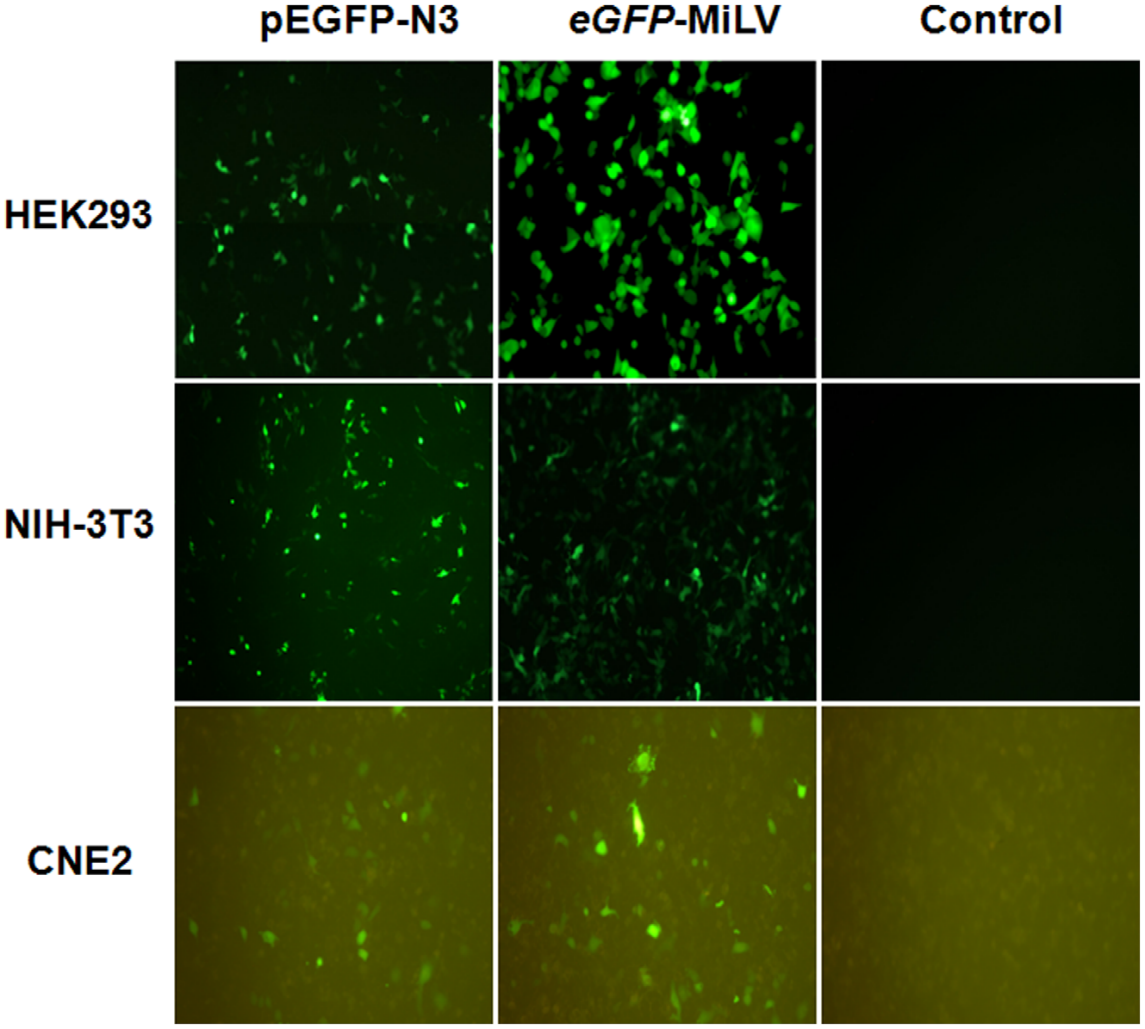
Expression of GFP in eukaryotic cells transfected with equal molar of *pEGFP-N3* plasmid or *eGFP*-MiLV. Cells were seeded in to six-well plates and transfected with MiLV or plasmid DNA (0.1 µM DNA/cm^2^ per plasmid). The transfection efficiency (expression of GFP) was evaluated 48 h after the transfection.

### Immunization of BALB/c Mice and Cell-mediated Immune Response

Forty micrograms *eGFP*-MiLV and *pEGFP-N3* plasmid were precipitated on 20 ml of 1 µm gold beads, respectively, according to the instructions of manufacturer (BioRad, USA). A suspension of gold beads carrying the DNA was made in 99.5% ethanol and the suspension was used to coat a 50 cm of tefzel tubing. The tubing was cut into 12.7 mm pieces and stored at −20°C before being used for gene gun immunizations. Then, 10-week-old female BALB/c mice were immunized with *eGFP*-MiLV and *pEGFP-N3* plasmid using a gene gun (HeliosTM, BioRad, USA). Three groups (Blank, MiLV and plasmid), each containing eight rodents, were vaccinated 4 times with 2 week intervals (0, 2, 4 and 6 weeks). The primary immunization (day 0) was performed with four cartridges, and the following immunizations were each performed using two cartridges. Blood samples were collected by orbital puncture of two individuals from each group every second day during the first 14 days, and at later time-points from all mice at days 28, 42 and 56, before doing the gene gun immunization.

**Figure 3:**
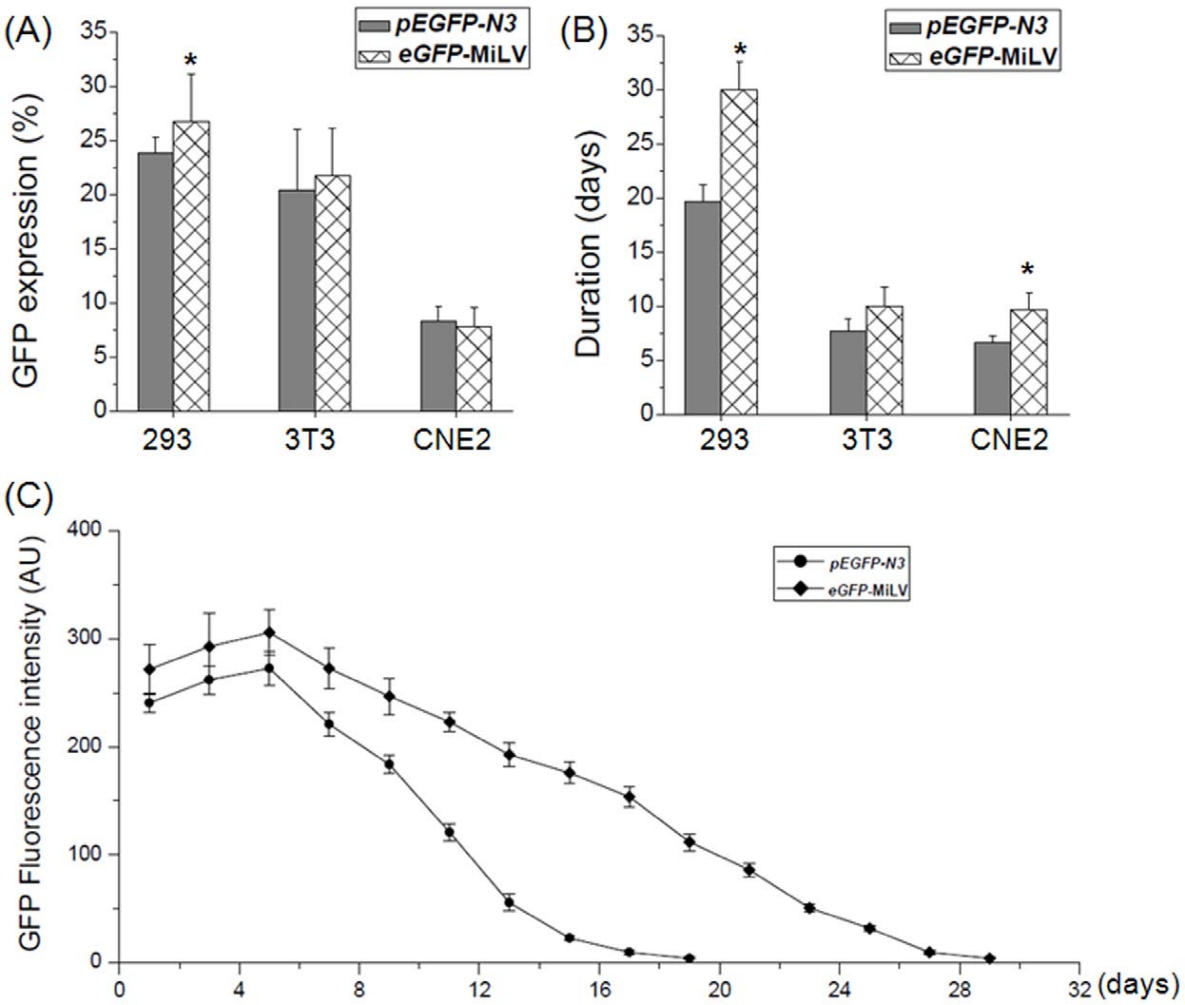
Expression ratio and duration of GFP in different eukaryotic cells transfected with equal molar of *pEGFP-N3* plasmid or *eGFP*-MiLV. A: Average ratio of GFP+ cells in HEK 293, NIH 3T3, CNE2 cells upon transfection with 0.1 µM DNA/cm^2^ *pEGFP-N3* plasmid or *eGFP*-MiLV for 48 h; B: The duration of GFP expression in HEK 293, NIH 3T3, CNE2 cells; C: The GFP fluorescence intensity AU (arbitrary units) of HEK 293 cells after transfected with 0.1 µM DNA/cm^2^ *pEGFP-N3* plasmid or *eGFP*-MiLV. Data are representative of at least three experiments. *p<0.05.

### Measurement of Pro-inflammatory Cytokines in the Blood

At 2 h after injection of 40 µg *eGFP*-MiLV and *pEGFP-N3* in lipoplex form (1∶1.5, g/ml, in a total volume of 50 µl) into the tail vein, the blood was collected by saphenous venepuncture. Blood samples were allowed to coagulate at 4°C for 4 h and then centrifuged at 4000×g for 10 min. Serum was collected, diluted with PBS and kept at −80°C until analysis. The concentrations of tumor necrosis factor (TNF)-α, interleukin (IL)-6 and IL-12 were determined using enzyme-linked immunosorbent assay (ELISA) kits.

**Figure 4:**
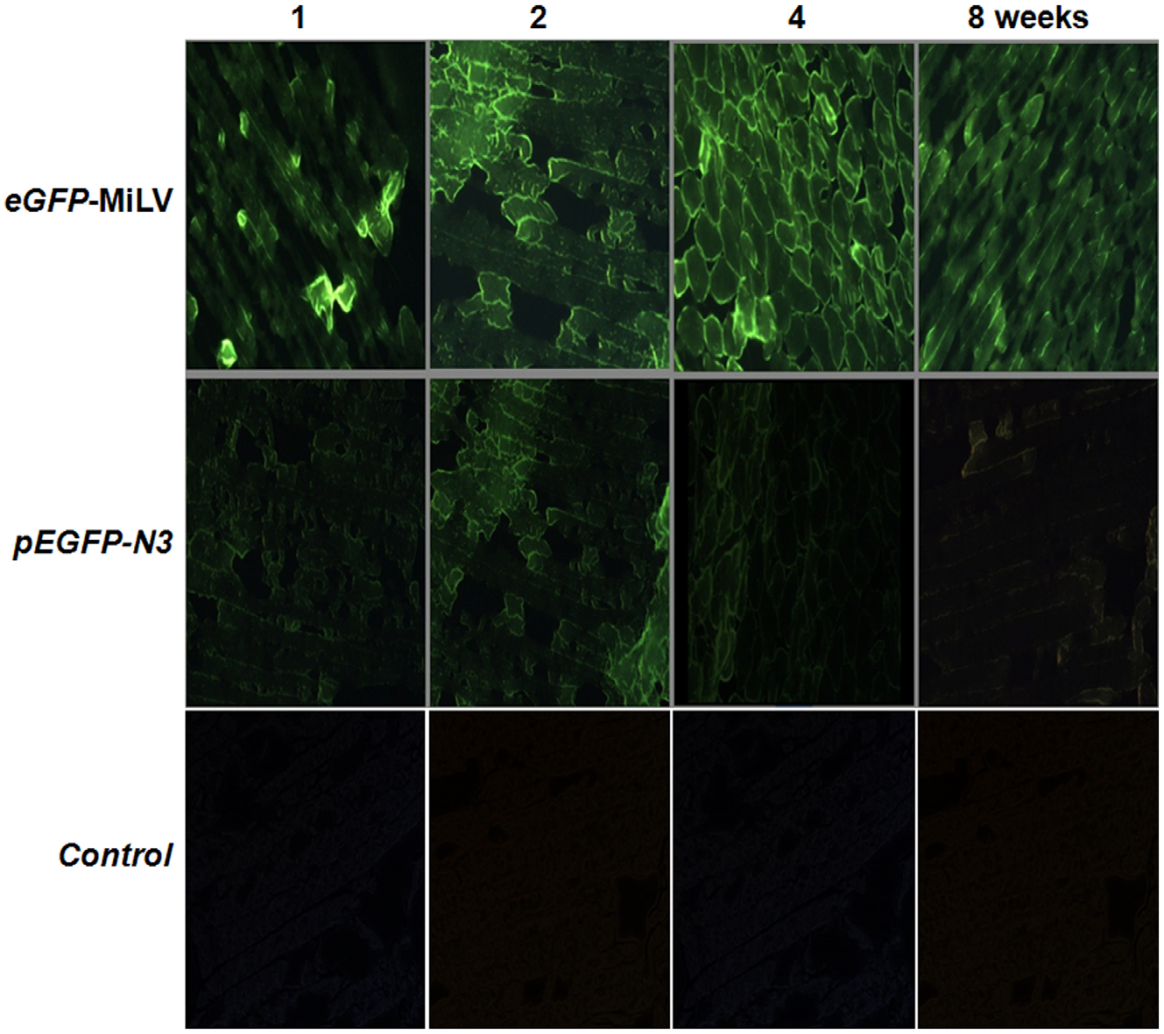
Expression of transgene of *eGFP-*MiLV and *pEGFP-N3* plasmid in mice muscle. After intramuscular injection, at least one mouse of each group was killed per week to detect the GFP expression. The muscle sections (5 µm) were observed by fluorescence microscope. The control group was injected with PBS. 1, 2, 4, 8 represents the weeks after intramuscular injection. The fluorescence of *eGFP-MiLV* lasted more than two months in mouse muscle.

### Immunoblot Analysis

The immunoblot assays were performed as described previously [18]. In brief, cells were lysed in buffer (1% Nonidet P-40, 20 mM Tris-HCl (pH 7.6), 0.15 M NaCl, 3 mM EDTA, 3 mM ethylene glycol tetraacetic acid (EGTA), 1 mM phenylmethylsulfonyl fluoride, 2 mM sodium vanadate, 20 mg/ml aprotinin, and 5 mg/ml leupeptin). The lysates were purified initially by centrifugation and denatured by boiling in Laemmli buffer, separated on 8% sodium dodecyl sulfate polyacrylamide gel electrophoresis (SDS-PAGE), and electrophoretically transferred to a nitrocellulose membrane. Following blocking with 5% non-fat milk at room temperature for 2 h, the membrane was incubated with the patient’s EBV-TK serum at 1∶1000 dilution overnight at 4°C. Membranes were then incubated with a 1∶5000 dilution of horseradish peroxidase conjugated secondary antibodies for 1 h at room temperature, and detected with the Western Lightning Chemiluminescent detection reagent (Perkin-Elmer Life Sciences, Wellesley, MA).

### *In vivo* Treatment Efficacy of MiLV

To evaluate the *in vivo* treatment efficacy of MiLV, 1×10^7^ B95-8 cells transfected with equal mole of *MEKK1-MiLV* or pCMV/MEKK1 plasmid were inoculated subcutaneously into both flanks of 10-week-old male BALB/c nude mice (8 mice for each treatment group). When tumors had become palpable (7–10 days later), they were treated with a single intraperitoneal injection of NaB (500 µl of 50 mM sodium butyrate in PBS) and intraperitoneal injection of GCV (100 mg/kg twice a day for 5 days). Tumor size was monitored by measuring the length and width with calipers, and volumes were calculated with the formula: (*L*×*W* ^2^) ×0.5, where *L* is length and *W* is width of each tumor. When tumors became extremely large (greater than 1 cm^3^) or the mice appeared ill, mice were sacrificed by cervical dislocation, and the tumors were excised and weighed.

**Figure 5:**
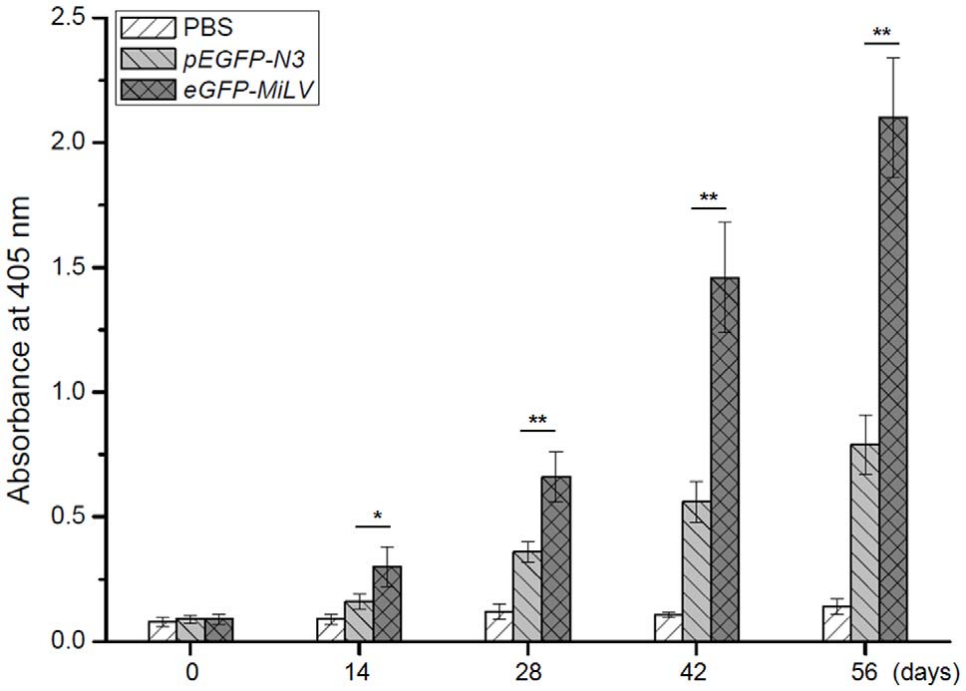
Mean ELISA values of GFP from equal weight (40 µg ) *eGFP*-MiLV and *pEGFP-N3* plasmid immunized mice. The ELISA was performed with mouse sera from individual animals of different vaccination groups. Blood samples were taken and prepared at the indicated time-points before each *eGFP*-MiLV or *pEGFP-N3* plasmid vaccination, and analyzed for reactivity against the GFP (the antigen). * p<0.05; ** p<0.01.

### Statistical Analysis

Statistical comparisons of differences between treatments were made by use of the paired *t* test. A p-value of <0.05 was considered to be statistically significant. The statistical analyses were performed using SPSS 17.0 for Windows.

**Figure 6:**
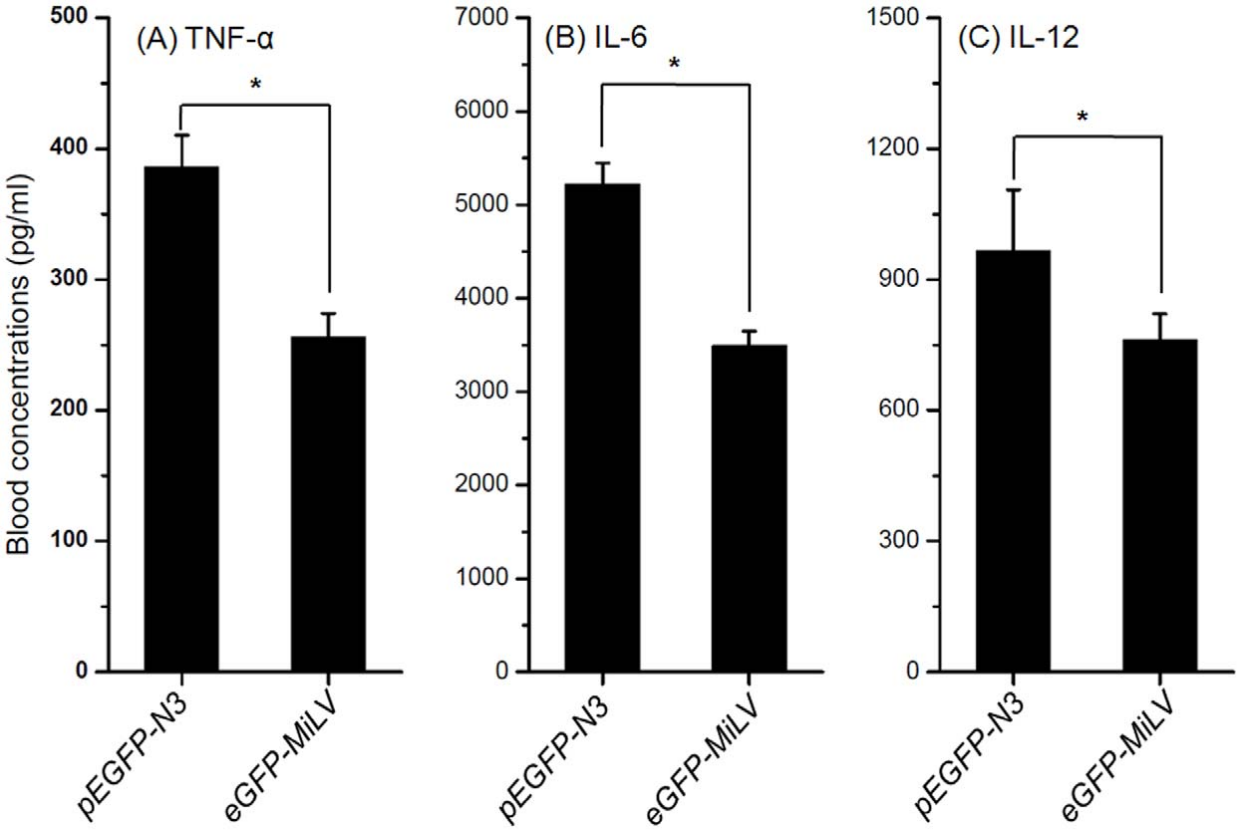
Effect of DNA-induced pro-inflammatory cytokines on GFP in the blood after intravenous injection *eGFP*-MiLV and *pEGFP-N3* plasmid. Mice received an intravenous injection of 40 µg *eGFP*-MiLV and *pEGFP-N3* plasmid. At 2 h after injection, the levels of TNF-α, IL-6 and IL-12 in blood were measured. The results are expressed at the mean ± SD of three mice. * p<0.05 compared to the *pEGFP-N3* group.

## Results

### Characteristics of MiLV

Compared with its progenitor *pEGFP-N3* plasmid (4.7 kb) which contains antibiotic makers and other bacterial originated genes, the *eGFP*-MiLV (1.7 kb) is only about one third the size (Figure 1). MiLV and PCR fragment were incubated with Exonuclease III to investigate the resistance of MiLV to *in vitro* degradation. The results indicated that the half-life of *eGFP*-MiLV was 10 to 15 folds greater than the DNA fragment alone (data not shown). More than 85% of MiLV were resistant against exonuclease digestion after 2 h. MTT assay showed that the viability of HEK 293 cells transfected with *eGFP*-MiLV (95.8±0.70%) were significantly (p<0.05) higher than that transfected with the pEGFP-N3 plasmid (92.8±0.67%). There was no significant (p>0.05) difference between cytostatic effects of MiLV or plasmid (data not shown).

**Figure 7:**
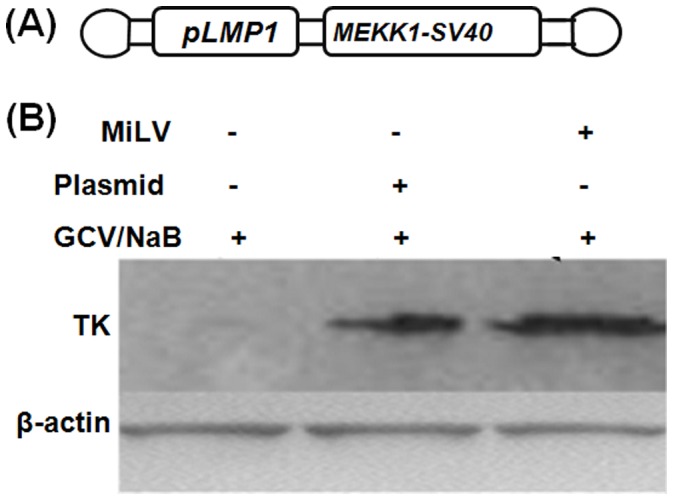
Expression of TK in B95-8 cell transfected by use of *MEKK1*-MiLV or *pCMV/MEKK1* plasmid. A: The structure of *MEKK1-MiLV*. The *pLMP1* resulted in MEKK1 being expressed only in EBV positive cells. B: B95-8 cells were transfected by equal molar of *MEKK1*-MiLV or *pCMV/MEKK1* plasmid for 24 h, and then treated with 1 mM NaB for 18 h, followed by treatment with 100 µg/ml GCV for 3 days. The TK expression was detected by immunoblot analysis with a patient’s EBV-TK serum. The β-actin protein levels served as the loading controls.

### *In vitro* GFP Transfection of MiLV and Plasmid

Three eukaryotic cell lines (HEK 293, NIH 3T3 and CNE2) were selected to compare the *in vitro* transfection efficiency of *eGFP*-MiLV and its corresponding plasmid *pEGFP-N3*. Green fluorescent protein was monitored 48 h later after transfection. When cells were transfected with equal molar concentrations of plasmid or *eGFP*-MiLV, transfection efficiencies were comparable (Figure 2). This result was confirmed by flow cytometry (Figure 3 A). In HEK 293 cells, *eGFP*-MiLV resulted in significantly (p<0.05) greater efficiency of transfection of GFP than the *pEGFP-N3*. The efficiency of transfection of *eGFP-MiLV* was as great as 30%. The efficiency of transfection of GFP of the two vectors was comparable in NIH 3T3 and CNE2 cell line. Duration of expression of GFP in cells transfected with *eGFP*-MiLV was significantly (p<0.05) greater than the duration of expression of GFP in both HEK 293 and CNE2 cells transfected with the *pEGFP-N3* plasmid, while comparable in NIH 3T3 cells (Figure 3 B). Expression of GFP lasted for nearly one month in HEK 293 cells when transferred with *eGFP*-MiLV, while the *pEGFP-N3* plasmid lasted for only about 20 days (Figure 3 C). This result suggests that the eGFP gene was more stable in eukaryotic cells when transfected by MiLV than cells transfected by use of a plasmid.

**Figure 8:**
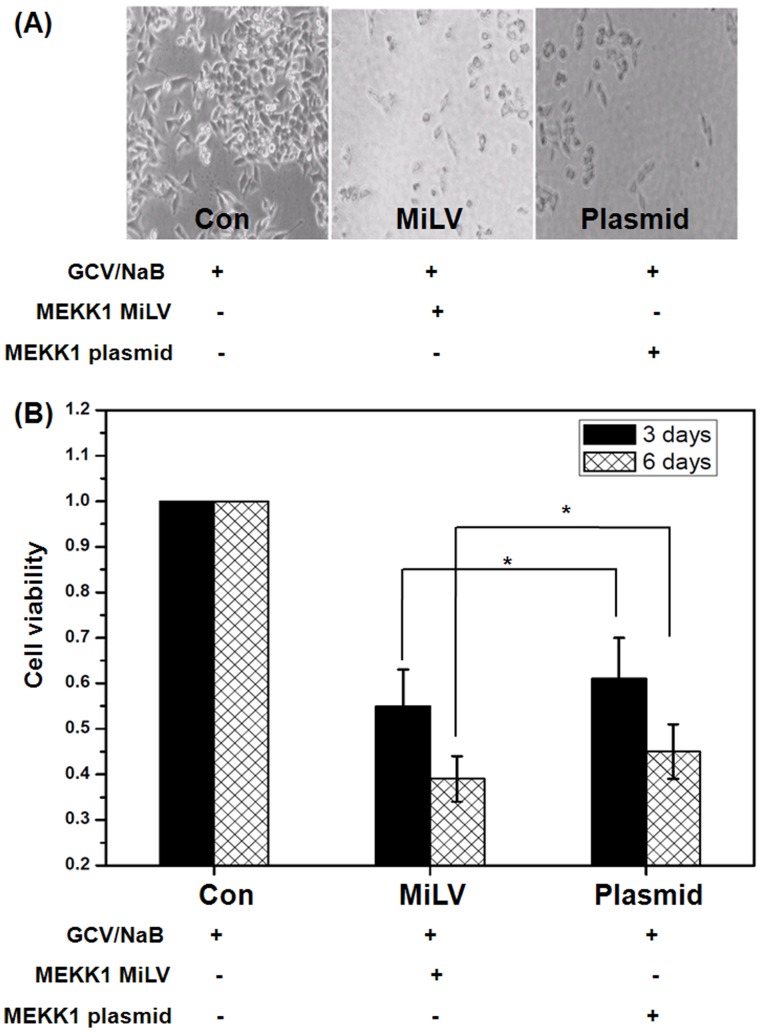
Relative viability of B95-8 cells transfected by *MEKK1*-MiLV or *pCMV/MEKK1* plasmid then incubated with GCV/NaB. Control was treated with GCV/NaB; MiLV group was transfected with *MEKK1*-MiLV and then treated with GCV/NaB; Plasmid group was transfected with *pCMV/MEKK1* plasmid and then treated with GCV/NaB. (A): The phenotypic change of B95-8 cells. Twenty four hours after transfection, cells were treated with 1 mM NaB for 18 h followed by 100 µg/ml GCV for 3 days. (B): MTT detected relative viability of B95-8 cells. The values are the mean of 3 separate experiments with error bars representing the standard deviations. The MEKK1 transfection enhanced the sensitivity of B95-8 cells to GCV/NaB. The *MEKK1*-MiLV transfected B95-8 cells were more sensitive (p<0.05, paired *t* test) to GCV/NaB than that of *pCMV/MEKK1* plasmid. * p<0.05.

### *In vivo* GFP Expression of MiLV and Plasmid

To assess *in vivo* expression of the GFP gene, 20 µg *eGFP*-MiLV or 60 µg *pEGFP-N3* plasmid (equal molar) was injected into a hind leg of mice. After durations of 1, 2, 4 or 8 weeks after intramuscular injection, at least one mouse of each group was euthanized. Green fluorescence was detected in muscle of mice more than two months after injection of *eGFP*-MiLV. Maximum fluorescence was observed 2–4 weeks after injection (Figure 4). However, there was just limited fluorescence observed 4 weeks after injection with the *pEGFP-N3* plasmid. The results suggested that MiLV is a more stable vector than plasmid for *in vivo* gene transfection. Transfection efficiency was quantified by measuring the fluorescence in muscle 2 weeks after injection. Fluorescence intensity in leg muscle of mice injected with *eGFP-MiLV* was significantly greater (3.2 fold, p<0.05, n = 3 for each group) than in the muscle of mice injected with the *pEGFP-N3* plasmid.

**Figure 9:**
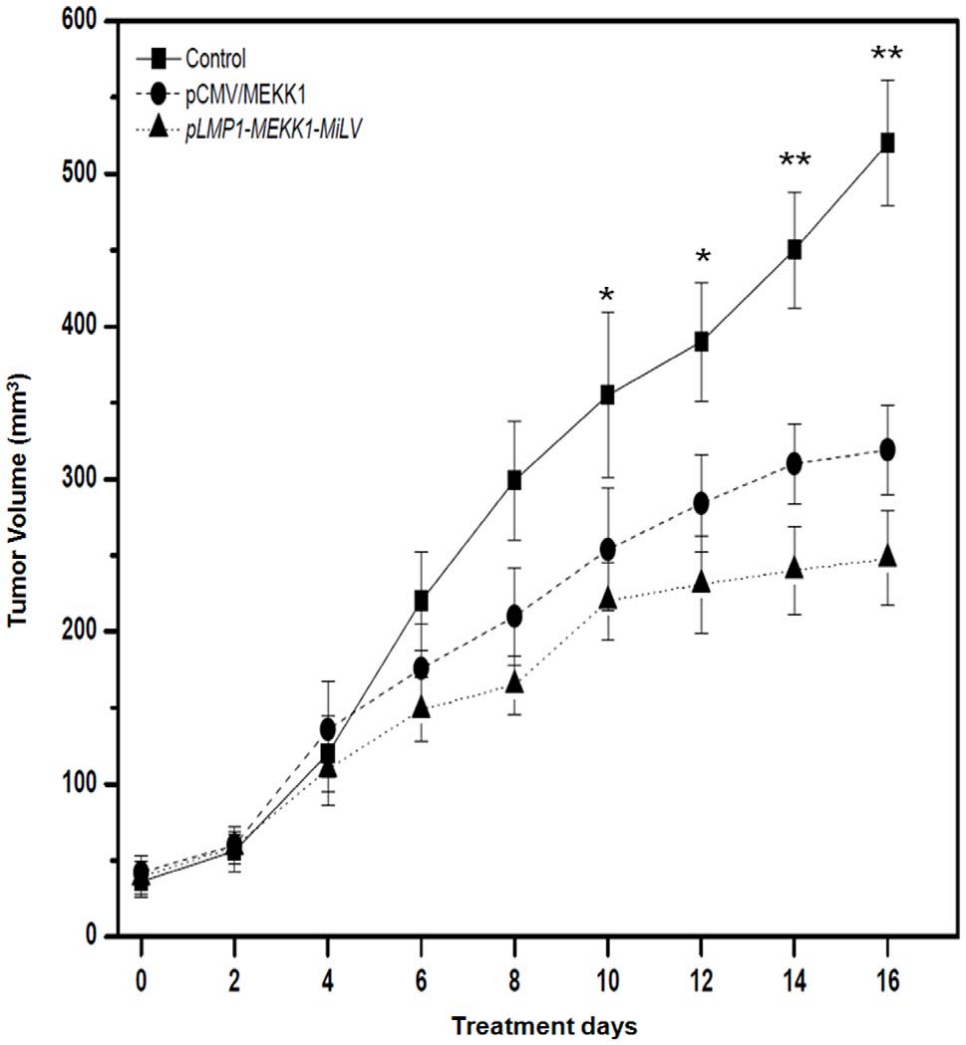
Inhibition of nasopharyngeal carcinoma growth in athymic nude mice with NaB/GCV treatment combined with *MEKK1*-MiLV or *pCMV/MEKK1* plasmid. The mice (8 mice per group) were inoculated with B95-8 cells transfected with equal mole of *MEKK1-*MiLV or *pCMV/MEKK1* plasmid. Then they were treated with single intraperitoneal injection of NaB (500 µl of 50 mM sodium butyrate in PBS) and intraperitoneal injection of GCV (100 mg/kg twice a day for 5 days). The cancer growth was monitored every 2 days using calipers. * p<0.05; ** p<0.01.

To compare the immune responses to encoded antigen of plasmid and MiLV, the *eGFP-MiLV* and *pEGFP-N3* plasmid were precipitated on gold particles and coated onto tefzel-tubings before gene gun immunization. Antibodies directly against the encoded antigen (GFP) were examined by ELISA in mouse sera taken at the indicated time-points after the primary immunization. Both the MiLV and plasmid group showed a positive antibody response towards the GFP antigen in serum samples taken 2 weeks after the first immunization. Mice immunized with MiLV showed a significantly (p<0.05) higher antibody response after the second immunization compared with the plasmid group (Figure 5). After four times immunization, the immune response of plasmid group is only about 38% of the MiLV group. It suggested that the gene delivery and expression efficiency of equal weight MiLV is significantly greater than that of plasmid.

### *In vivo* Inflammatory Response

The immunostimulatory activities of the *eGFP*-MiLV and *pEGFP-N3* formulations were tested by determining serum TNF-α, IL-6 and IL-12 levels after injection (40 µg DNA) for 2 h. Figure 6 shows the levels of TNF-α, IL-6 and IL-12 levels in blood at 2 h after injection of *eGFP*-MiLV and *pEGFP-N3*. The levels of TNF-α, IL-6 and IL-12 in *pEGFP-N3* group were 1.5-, 1.4-, and 1.2-fold higher than that in *eGFP*-MiLV group, respectively. The results revealed that MiLV, which contains much less CpG motifs than plasmid, reduces the *in vivo* inflammatory responses to gene delivery vector.

### Case Study: *MEKK1*-MiLV to EBV Positive Cells and Tumor

Because the results of the *in vitro* and *in vivo* proof of concept studies suggested that MiLV had good potential application prospects at gene therapy, the *MEKK1* gene, which can elevate the sensitivity of B95-8 cells to NaB/GCV and the expression of thymidine kinase (TK) gene [18], was chosen to construct *MEKK1-*MiLV (Figure 7 A). The *pLMP1* promoter was included to ensure that MEKK1 would only be expressed in cells that are EBV positive. The immunoblot results revealed that MEKK1 delivered by MiLV or plasmid could significantly enhance the TK expression in B95-8 cells (Figure 7 B), which was consistent with our previous study [18]. The results indicated that MEKK1 transfection efficiency of *MEKK1-*MiLV was at least comparable with pCMV/MEKK1 plasmid.

After being transfected with either *MEKK1-*MiLV or MEKK1 plasmid for 24 h, B95-8 cells were treated with 1 mM NaB for 18 h followed by exposure to 100 µg/ml GCV for 3 days. Nuclei of cells transfected with the MEKK1 plasmid or *MEKK1-*MiLV became smaller, elongated and fusiform (Figure 8 A). The relative viability of *MEKK1-*MiLV transfected cells (Figure 8 A, MiLV group ) was less than MEKK1 plasmid transfected cells (Figure 8 A, Plasmid group). This result was confirmed by the results of the MTT assay. B95-8 cells transfected with *MEKK1-*MiLV were significantly (p<0.05, paired *t* test) more sensitive than cells transfected with the plasmid to co-exposure to GCV/NaB for 3 or 6 days (Figure 8 B). Thus, it was concluded that transfection with MEKK1 could enhance the sensitivity of EBV positive cells to GCV/NaB, particularly when MEKK1 was transferred by MiLV.

We tested the effects of MEKK1 gene delivered by MiLV or plasmid on established nasopharyngeal carcinoma growth in mice. As shown in Figure 9, tumor cells transfected with *MEKK1-*MiLV or *pCMV/MEKK1* plasmid both significantly suppressed the growth of B95-8 subcutaneous tumors when compared with that of control (not transfected) (p<0.01), suggesting that MEKK1 gene could enhance the *in vivo* sensitivity of EBV positive tumor cells to GCV/NaB. Furthermore, the tumor volumes were significantly (p<0.05) reduced in the group treated with *MEKK1-*MiLV compared with the group treated with *pCMV/MEKK1* plasmid. These results suggested that the *MEKK1-MiLV* has a more favorable antitumor effect than plasmid *in vivo*.

## Discussion

The design and optimization of the expression system is a major part of the development of successful gene therapy [19]. One reasonable approach to enhance the efficiency of transfection is to remove the non therapeutic genes from the plasmid, especially immunostimulatory CpG motifs that originate in bacteria [20]. In the present study, the ligation product was used directly as the template for PCR amplification. The vector was duplicated at the end of each PCR cycle. After the vector was purified using a PCR cleanup kit and ensured by DNA sequencing, it could be used for *in vitro* and *in vivo* experiments. A possible concern about using PCR amplification for MiLV is that errors occur during amplification. Two or more polymerases (such as Taq and Pyrobest polymerases) with different fidelities can reduce these errors. Furthermore, DNA sequencing after PCR amplification was used to ensure that there was no mutation for DNA-MiLV transfection. The PCR-amplified MiLV has several significant advantages over plasmid DNA and other similar gene delivery vectors.

The MiLV is safer than plasmids and other vectors. The MiLV reduces numbers of inflammatory unmethylated CpG motifs which are contained in the skeleton of plasmid. Unmethylated CpG dinucleotides, or CpG motifs, which are uncommon in mammalian DNA, stimulate immune cells through Toll-like receptor 9 (TLR9). This recognition results in the production of pro-inflammatory cytokines, especially when DNA is administered as a DNA/cationic liposome complex [21], [22]. Our results revealed that *in vivo* inflammatory response levels (levels of TNF-α, IL-6 and IL-12) of *pEGFP-N3* group were significantly higher than in the *eGFP*-MiLV group, which elevates the *in vivo* gene delivery safety of MiLV. In addition, the PCR-amplified MiLV can avoid contaminations of bacterial origin during plasmid extraction [15]. Minicircle DNA has been approved to be an efficient DNA vector which is a double-stranded circular DNA with reduced size [7], [23]. The *in vitro* and *in vivo* efficiency of gene transfer for MiLV and minicircle DNA were not compared in the present study. However, the MiLV could avoid contaminations of the bacterial originated endotoxin such as LPS which might be involved during the production processes of minicircle DNA [5], [24]. Furthermore, MiLV might reduce the chances of chromosomal integration into mammalian genomes than that of plasmid DNA, which may cause toxic adverse effects [25]. The results of previous studies have shown that transcriptionally active linear DNA fragments were not well integrated into the genome and remained predominately extrachromosomal in mammalian organs [26], [27]. It is reasonable to assume that genes delivered by MiLV are less likely to be integrated into the genome because the expression cassette flanked by two caps is linear fragment. Therefore, adverse effects of MiLV being integrated into the genome might be less than those caused by use of the plasmid.

MiLV improves the gene delivery efficiency. The results of *in vitro* experiments revealed that efficiencies of transfection of the *eGFP* gene delivered to eukaryotic cell lines such as HEK 293, NIH 3T3 and CNE2 by MiLV were at least comparable or greater than the plasmid. In order to efficiently transfect cells, gene delivery vectors have to pass through nuclepores; which favor small and actively transported molecules [6], [28]. Previous studies indicated that DNA fragments greater than 1 kb remain in the cytoplasm rather than entering the nucleus [29]. Therefore, larger DNA molecules have less opportunity to enter the nucleus. The improvement of gene transfection efficiency by MiLV might be due to the smaller size. Furthermore, pro-inflammatory cytokines, which are stimulated by unmethylated CpG motifs as mentioned above, have been reported to reduce the transgene expression in later periods of gene transfer [30], [31]. MiLV, which reduces the *in vivo* inflammatory response as compared to plasmid, would elevate the gene transfection efficiency. In addition, the CpG motifs can be the target for methylation by DNA methyltransferases. About 70% of CpG sites in the CMV enhancer were methylated at days 7 after intramuscular injection of adenoviral vectors [32]. Methylation is a major mechanism responsible for the reduced gene expression in eukaryotic cells [33]. One recent study revealed that the deletion of CpG motifs in plasmid improved the duration of *in vivo* transgene expression when administered as a DNA/polymer complex [34]. Collectively, the greater *in vitro* and *in vivo* gene delivery efficiency of the MiLV than plasmid in the present study might be due to the reduction of bacterial CpG motifs as well as its small size.

Stability is another important factor affecting expression of transgenes. In the present study, new ODN caps were designed according to the D loop of tRNA in eukaryotic cells. The results of *in vitro* digestion experiments revealed that the cap could protect the vector from exonuclease effectively (85% of MiLV were resistant against exonuclease digestion for 2 h). Furthermore, the duration of GFP expression *in vitro* or *in vivo* that had been transfected by use of MiLV was significantly greater than that transfected by use of the plasmid. Fluorescence was quenched after 4 weeks in mice transfected by using the plasmid; while in mice transfected with MiLV, fluorescence lasted for more than 2 months. In addition to small molecules enhancing the transfection efficiency of MiLV, another reason for lower stability of plasmid is that the presence of CpG motifs, which triggers the induction of inflammatory cytokines upon administration to animals [35]. This feature is a drawback for the sustained expression of transgenes incorporated using plasmid. In the case of the MiLV, the continuous expression of the foreign genes might be due to the fact that almost all nontherapeutic sequences have been removed. Therefore, mice immunized with MiLV showed a significantly (p<0.05) lower pro-inflammatory response compared with the plasmid group. Further studies will be needed to demonstrate more detailed reasons for the prolonged transgene expression of MiLV.

EBV is a ubiquitous human herpes virus that is associated with variety of human malignancies, including nasopharyngeal carcinoma (NPC), Burkitt’s lymphoma (BLs), T cell lymphoma and gastric carcinoma [36]. Nearly 100% of NPCs and 90% of BLs contain EBV episomes [37]. Our previous study indicated that the constitutive activation of MEKK1 can increase the sensitivity of EBV positive cells to GCV/NaB via a TK-dependent mechanism [18]. In the present study, the cells transfected with *MEKK1*-MiLV were more sensitive to GCV/NaB than that transfected with plasmid. Furthermore, our results showed that tumor cells transfected with *MEKK1-*MiLV or *pCMV/MEKK1* plasmid had significantly smaller B95-8 subcutaneous tumors when compared with that of the control. The *in vivo* antitumor efficacy of *MEKK1-*MiLV was significantly greater than its corresponding plasmid. These results also suggested that interfering with the MEKK1 signaling pathway may be a useful therapeutic strategy to enhancing the sensitivity of EBV-positive tumor cells to GCV/NaB.

In summary, this study provides proof of the efficacy of a safer gene delivery vector with satisfactory transfection efficiency both *in vitro* and *in vivo*. This PCR generated vector does not require bacteria for production. Therefore, it removes the possibility of LPS contamination during plasmid preparation. These advantages combined with the optimized biological safety encourage further development and the preferential use of this new vector type in clinical gene therapy studies.

## References

[1] Schmidt-Wolf GD, Schmidt-Wolf IGH (2003) Non-viral and hybrid vectors in human gene therapy: an update. Trends Mol Med 9: 67–72. 12615040 10.1016/s1471-4914(03)00005-4

[2] Glover DJ, Lipps HJ, Jans DA (2005) Towards safe, non-viral therapeutic gene expression in humans. Nat Rev Genet 6: 299–310. 15761468 10.1038/nrg1577

[3] Nishikawa M, Hashida M (2002) Nonviral approaches satisfying various requirements for effective *in vivo* gene therapy. Bio Pharm Bull 25: 275–283. 11913519 10.1248/bpb.25.275

[4] Coban C, Ishii KJ, Gursel M, Klinman DM, Kumar N (2005) Effect of plasmid backbone modification by different human CpG motifs on the immunogenicity of DNA vaccine vectors. J Leukoc Biol 78: 647–655. 15961575 10.1189/jlb.1104627

[5] Darquet AM, Rangara R, Kreiss P, Schwartz B, Naimi S, et al (1999) Minicircle: an improved DNA molecule for *in vitro* and *in vivo* gene transfer. Gene Ther 6: 209–218. 10435105 10.1038/sj.gt.3300816

[6] Johansson P, Lindgren T, Lundstrom M, Holmstrom A, Elgh F, et al (2002) PCR-generated linear DNA fragments utilized as a hantavirus DNA vaccine. Vaccine 20: 3379–3388. 12213408 10.1016/s0264-410x(02)00265-7

[7] Chang CW, Christensen LV, Lee M, Kim SW (2008) Efficient expression of vascular endothelial growth factor using minicircle DNA for angiogenic gene therapy. J Control Release 125: 155–163. 18063165 10.1016/j.jconrel.2007.10.014 PMC2677388

[8] Reyes-Sandoval A, Ertl HCJ (2004) CpG methylation of a plasmid vector results in extended transgene product expression by circumventing induction of immune responses. Mol Ther 9: 249–261. 14759809 10.1016/j.ymthe.2003.11.008

[9] Chen ZY, He CY, Kay MA (2005) Improved production and purification of minicircle DNA vector free of plasmid bacterial sequences and capable of persistent transgene expression *in vivo* . Hum Gene Ther 16: 126–131. 15703495 10.1089/hum.2005.16.126

[10] Schakowski F, Gorschluter M, Junghans C, Schroff M, Buttgereit P, et al (2001) A novel minimal-size vector (MIDGE) improves transgene expression in colon carcinoma cells and avoids transfection of undesired DNA. Mol Ther 3: 793–800. 11356084 10.1006/mthe.2001.0322

[11] Machelska H, Schroff M, Oswald D, Binder W, Sitte N, et al (2009) Peripheral non-viral MIDGE vector-driven delivery of beta-endorphin in inflammatory pain. Mol Pain 5: 72. 20003437 10.1186/1744-8069-5-72 PMC2797781

[12] Lopez-Fuertes L, Perez-Jimenez E, Vila-Coro AJ, Sack F, Moreno S, et al (2002) DNA vaccination with linear minimalistic (MIDGE) vectors confers protection against Leishmania major infection in mice. Vaccine 21: 247–257. 12450700 10.1016/s0264-410x(02)00450-4

[13] Zheng C, Juhls C, Oswald D, Sack F, Westfehling I, et al (2006) Effect of different nuclear localization sequences on the immune responses induced by a MIDGE vector encoding bovine herpesvirus-1 glycoprotein D. Vaccine. 24: 4625–4629. 10.1016/j.vaccine.2005.08.046 16154243

[14] Schakowski F, Gorschluter M, Buttgereit P, Marten A, Lilienfeld-Toal MV, et al (2007) Minimal size MIDGE vectors improve transgene expression *in vivo* . In Vivo 21: 17–23. 17354609

[15] Hofman CR, Dileo JP, Li Z, Li S, Huang L (2001) Efficient *in vivo* gene transfer by PCR amplified fragment with reduced inflammatory activity. Gene Ther 8: 71–74. 11402304 10.1038/sj.gt.3301373

[16] Hirata K, Nishikawa M, Kobayashi N, Takahashi Y, Takakura Y (2007) Design of PCR-amplified DNA fragments for *in vivo* gene delivery: Size-dependency on stability and transgene expression. J Pharm Sci 96: 2251–2261. 17387694 10.1002/jps.20879

[17] Xu X, Yang Z, Liu Q, Wang Y (2010) *In vivo* fluorescence imaging of muscle cell regeneration by transplanted EGFP-labeled myoblasts. Mol Ther 18: 835–842. 20125125 10.1038/mt.2010.3 PMC2862520

[18] He YW, Cai SH, Zhang G, Li XQ, Pan LT, et al (2008) Interfering with cellular signaling pathways enhances sensitization to combined sodium butyrate and GCV treatment in EBV-positive tumor cells. Virus Res 135: 175–180. 18455826 10.1016/j.virusres.2008.03.012

[19] Esin S, Batoni G, Kallenius G, Gaines H, Campa M, et al (1996) Proliferation of distinct human T cell subsets in response to live, killed or soluble extracts of Mycobacterium tuberculosis and Mycoavium. Clin Exp Immunol 104: 419–425. 9099925 10.1046/j.1365-2249.1996.d01-691.x PMC2200458

[20] Mitsui M, Nishikawa M, Zang L, Ando M, Hattori K, et al (2009) Effect of the content of unmethylated CpG dinucleotides in plasmid DNA on the sustainability of transgene expression. J Gene Med 11: 435–443. 19291673 10.1002/jgm.1317

[21] Yoshida H, Nishikawa M, Yasuda S, Mizuno Y, Takakura Y (2008) Cellular activation by plasmid DNA in various macrophages in primary culture. J Pharm Sci 97: 4575–4585. 18228575 10.1002/jps.21302

[22] Yasuda K, Ogawa Y, Yamane I, Nishikawa M, Takakura Y (2005) Macrophage activation by a DNA/cationic liposome complex requires endosomal acidification and TLR9-dependent and -independent pathways. J Leukoc Biol 77: 71–79. 15496451 10.1189/jlb.0204089

[23] Zhang X, Epperly MW, Kay MA, Chen ZY, Dixon T, et al (2008) Radioprotection *in vitro* and *in vivo* by minicircle plasmid carrying the human manganese superoxide dismutase transgene. Hum Gene Ther 19: 820–826. 18699723 10.1089/hum.2007.141 PMC2914206

[24] Chen ZY, He CY, Ehrhardt A, Kay MA (2003) Minicircle DNA vectors devoid of bacterial DNA result in persistent and high-level transgene expression *in vivo* . Mol Ther 8: 495–500. 12946323 10.1016/s1525-0016(03)00168-0

[25] Nakai H, Montini E, Fuess S, Storm TA, Meuse L, et al (2003) Helper-independent and AAV-ITR-independent chromosomal integration of double-stranded linear DNA vectors in mice. Mol Ther 7: 101–111. 12573623 10.1016/s1525-0016(02)00023-0

[26] Chen ZY, Yant SR, He CY, Meuse L, Shen S, et al (2001) Linear DNAs concatemerize *in vivo* and result in sustained transgene expression in mouse liver. Mol Ther 3: 403–410. 11273783 10.1006/mthe.2001.0278

[27] Kameda S, Maruyama H, Higuchi N, Nakamura G, Iino N, et al (2003) Hydrodynamics-based transfer of PCR-amplified DNA fragments into rat liver. Biochem Biophys Res Commun 309: 929–936. 13679063 10.1016/j.bbrc.2003.08.087

[28] Ludtke JJ, Zhang GF, Sebestyen MG, Wolff JA (1999) A nuclear localization signal can enhance both the nuclear transport and expression of 1 kb DNA. J Cell Sci 112: 2033–2041. 10341220 10.1242/jcs.112.12.2033

[29] Hagstrom JE, Ludtke JJ, Bassik MC, Sebestyen MG, Adam SA, et al (1997) Nuclear import of DNA in digitonin-permeabilized cells. J Cell Sci 110: 2323–2331. 9378781 10.1242/jcs.110.18.2323

[30] Kako K, Nishikawa M, Yoshida H, Takakura Y (2008) Effects of inflammatory response on *in vivo* transgene expression by plasmid DNA in mice. J Pharm Sci 97: 3074–3083. 18064709 10.1002/jps.21254

[31] Tan Y, Li S, Pitt BR, Huang L (1999) The inhibitory role of CpG immunostimulatory motifs in cationic lipid vector-mediated transgene expression *in vivo* . Hum Gene Ther 10: 2153–2161. 10498247 10.1089/10430349950017149

[32] Brooks AR, Harkins RN, Wang P, Qian HS, Liu P, et al (2004) Transcriptional silencing is associated with extensive methylation of the CMV promoter following adenoviral gene delivery to muscle. J Gene Med 6: 395–404. 15079814 10.1002/jgm.516

[33] Grewal SI, Moazed D (2003) Heterochromatin and epigenetic control of gene expression. Science 301: 798–802. 12907790 10.1126/science.1086887

[34] de Wolf HK, Johansson N, Thong AT, Snel CJ, Mastrobattista E, et al (2008) Plasmid CpG depletion improves degree and duration of tumor gene expression after intravenous administration of polyplexes. Pharm Res 25: 1654–1662. 18317886 10.1007/s11095-008-9558-7 PMC2440937

[35] Wilson KD, de Jong SD, Tam YK (2009) Lipid-based delivery of CpG oligonucleotides enhances immunotherapeutic efficacy. Adv Drug Deliv Rev 61: 233–242. 19232375 10.1016/j.addr.2008.12.014

[36] Li HP, Leu YW, Chang YS (2005) Epigenetic changes in virus-associated human cancers. Cell Res 15: 262–271. 15857581 10.1038/sj.cr.7290295

[37] Young LS, Rickinson AB (2004) Epstein-Barr virus: 40 years on. Nat Rev Cancer 4: 757–768. 15510157 10.1038/nrc1452

